# Developmental Maturation of the Cerebellar White Matter—an Instructive Environment for Cerebellar Inhibitory Interneurons

**DOI:** 10.1007/s12311-020-01111-z

**Published:** 2020-01-30

**Authors:** Anne Groteklaes, Carina Bönisch, Britta Eiberger, Andrea Christ, Karl Schilling

**Affiliations:** Anatomisches Institut, Anatomie & Zellbiologie, Rheinische Friedrich-Wilhelms-Universität, Nussallee 10, D-53115 Bonn, Germany

**Keywords:** Cerebellum, GABAergic interneurons, Basket cells, Stellate cells, Cerebellar glia, Pax2

## Abstract

In the developing cerebellum, the nascent white matter (WM) serves as an instructive *niche* for cerebellar cortical inhibitory interneurons. As their Pax2 expressing precursors transit the emerging WM, their laminar fate is programmed. The source(s) and nature of the signals involved remain unknown. Here, we used immunocytochemistry to follow the cellular maturation of the murine cerebellar WM during this critical period. During the first few days of postnatal development, when most Pax2 expressing cells are formed and many of them reach the cerebellar gray matter, only microglial cells can be identified in the territories through which Pax2 cells migrate. From p4 onward, cells expressing the oligodendrocytic or astrocyte markers, CNP-1, MBP or GFAP, started to appear in the nascent WM. Expression of macroglial markers increased with cerebellar differentiation, yet deep nuclei remained GFAP-negative at all ages. The progressive spread of maturing glia did not correlate with the exit of Pax2 cells from the WM, as indicated by the extensive mingling of these cells up to p15. Whereas sonic hedgehog-associated p75^NTR^ expression could be verified in granule cell precursors, postmitotic Pax2 cells are p75^NTR^ negative at all ages analyzed. Thus, if Pax2 cells, like their precursors, are sensitive to sonic hedgehog, this does not affect their expression of p75^NTR^. Our findings document that subsequently generated sets of Pax2 expressing precursors of inhibitory cerebellar interneurons are confronted with a dynamically changing complement of cerebellar glia. The eventual identification of fate-defining pathways should profit from the covariation with glial maturation predicted by the present findings.

## Introduction

The cerebellum is derived from two major germinative layers, both of which ultimately originate from the dorso-rostral neuroepithelium lining the fourth ventricle (for a recent review and further references, see [1]). In the mouse, this part of the neuroepithelium may be discerned on embryonic day nine, and it subsequently gives rise to the rhombic lip, from which excitatory (glutamatergic) cerebellar neurons originate [2 pp. 80f; 3, 4], and the ventricular zone, which generates all GABAergic neurons, including Purkinje cells, GABAergic nucleo-olivary projection neurons and inhibitory interneurons of deep cerebellar nuclei and of the cerebellar cortex [5–8].

As precursors of inhibitory interneurons translocate from the ventricular epithelium to their definitive positions, the deep cerebellar mass and the nascent white matter function not only as a mere conduit. As first pointed out by Zhang and Goldman [8], these cells divide while in transit. Once they become postmitotic, they may be recognized by their expression of Pax2 [9], which starts during the last cell division [10, 11]. Subsequent studies, primarily by Ketty Leto and Ferdinado Rossi, revealed that adequate differentiation of cerebellar inhibitory interneurons depends on their transit through the nascent white matter. In a series of elegant experiments, they documented that transplanted interneuron-precursors develop independently of the age of the donor animal from which they were taken. Rather, transplanted cells developed into exactly those types of GABAergic interneurons that were also formed by hosts cells transiting the prospective white matter at the time of transplantation [10; see also 7]. Together, these findings led to the view that within the deep cerebellar mass and nascent white matter (WM) of the cerebellar anlage, precursors of inhibitory cerebellar interneurons are confronted with signals that regulate their numeric expansion and program their final phenotypic differentiation [12, 13].

During the rather protracted transit of inhibitory interneuron precursors through the deep cerebellar mass and nascent white matter, this environment changes extensively: afferent (mossy and climbing fibers) and efferent (Purkinje cells and deep nuclei efferent) fibers, mature. Morphologically, this is reflected primarily by conspicuous changes of the glial cells making up the (prospective) white matter (for a review on macroglia, see [14]; for microglia, see [15, 16]).

To date, these changes have not been related directly to the development of cerebellar inhibitory interneurons (see, e.g., [17]). Here, we present results from a series of experiments aimed to describe some aspects of the cellular composition and maturation of the nascent cerebellar white matter in the early postnatal period, i.e., the period during which most cerebellar inhibitory interneurons are generated, migrate and differentiate through this “instructive niche” [13]. It is hoped that these data help to focus the search for factors that govern cerebellar inhibitory interneuron differentiation.

## Materials and Methods

### Animals and Tissue Preparation

We used wild type C57BL/6 mice and transgenic mice of the same background expressing a Pax2-GFP fusion protein (BAC line #30, [11, 18]. All animal handling was done in strict adherence to governmental (Directive 2010/63/EU of the European parliament and of the Council of 22 September 2010) and institutional animal care regulations.

For tissue preparation, newborn mice (p0) and mice aged 4, 6, 8, 15, or 28 days (p4, p6, p8, p15, p28) as well as adult (p56-p70) mice were deeply anesthetized by intraperitoneal injection of Ketamin/Xylazin (10:1 Ketamin:Xylazin in 0.9%NaCl, 0.1 ml/10 g body weight). Animals were then perfused through their left ventricle with phosphate-buffered saline (PBS, 150 mM NaCl, 10 mM NaH_2_PO_4_·H_2_O, pH 7.4), followed by perfusion with 4% paraformaldehyde (PFA) in PBS. After dissection, brains were postfixed in 2% PFA in PBS overnight. They were then transferred into tab water and rinsed for 1 week. Subsequently, they were dehydrated by passage through graded alcohols, transferred into xylol, and embedded in paraffin. Sagittal sections from the vermal and paravermal regions were cut with a microtome at 10 μm and mounted on superfrost plus glass slides (Thermo Scientific, Rockford, IL, USA).

### Immunofluorescence Staining

For immunostaining, sections were heated up to 60 degrees for 20 min to enhance their adhesion to the slides. They were then deparaffinized and rehydrated using xylol and a passage through a series of graded alcohols. Antigens were retrieved by boiling sections in citrate puffer (10 mM, pH 6.0) containing 0.05% (v/v) Tween 20 for 20 min. Subsequently, sections were permeabilized for 10 min with 0.5% Triton-X-100 in PBS. This last step was omitted if sections were to be stained for Iba-1. Free protein binding sides were blocked with blocking solution (0.2% gelatin in PBS) at room temperature for 2 h. Next, sections were incubated at 4 °C overnight with primary antibodies (for dilution used, suppliers and specificity, see Table 1) diluted in blocking solution. Subsequently, sections were washed three times in PBS and Alexa 488 and 546-tagged secondary antibodies (Thermo Fisher, Rockford, IL, USA) diluted 1:400 in blocking solution were applied for 2 h at room temperature. Finally, sections were washed in PBS, counterstained with Hoechst 33342 (1 μg/ml in PBS) and embedded with Dako fluorescence mounting medium (Dako, Glostrup, Denmark). For negative controls, sections were incubated without primary antibodies. For each age and each antigen analyzed, sections obtained from at least three different animals (of either sex) were studied, and at least 4 sections per animal were evaluated.

**Table 1:** Antibodies used

Antibody (species, supplier)	Dilution	Reference
CNP-1 (mouse, Synaptic Systems, Göttingen, Germany)	1:500	Braun et al. 1988
GFAP (mouse, Merck Millipore, Darmstadt, Germany)	1:1000	Eng, et al. 1985
GFP (chicken, Abcam, Cambridge, UK)	1:500	Kamitakahara et al. 2017
MBP (rabbit, Synaptic Systems, Göttingen, Germany)	1:500	Brunner et al. 1999
Phosphohiston H3 (rabbit,, Merck Millipore, Darmstadt, Germany)	1:500	Hendzel et al. 1997
Pax 2 (rabbit, Thermo Fisher, Rockford, IL, USA)	1:500	Weisheit et al. 2006
Anti-p75 NGF receptor (rabbit, Abcam, Cambridge, UK)	1:300	Küchler et al. 2010
Anti-Iba-1 (rabbit, Wako, Osaka, Japan)	1:500	Ahmed et al. 2007

### Microscopy and Image Processing

Fluorescent images were acquired with a Zeiss Axioskop and the following objectives: Plan Neofluar × 2.5, NA 0.075; Plan Apochromat × 10, NA 0.3; Plan Neofluar × 20, NA 0.5. They were recorded using a Cool snap camera (Visitron Systems; resolution of 1392 × 1040 pixels) and Axiovision software (Zeiss; release 4.82). Adobe Photoshop was used for the adjustment of image brightness and contrast (linear adjustments) and the arrangement of images.

## Results

### Developmental Distribution of Pax2-GFP Cells in the Cerebellar Anlage

The distribution of inhibitory interneuronal precursors in the cerebellar anlage has been described previously by [9], who also established that Pax2 is a specific marker for these immature cells. Further, Weisheit et al. [11] verified that Pax2-GFP expression faithfully replicates expression of the cognate Pax2 gene. Therefore, we refer to inhibitory interneuronal precursors identified by Pax2-GFP-expression as Pax2 cells.

**Fig. 1:**
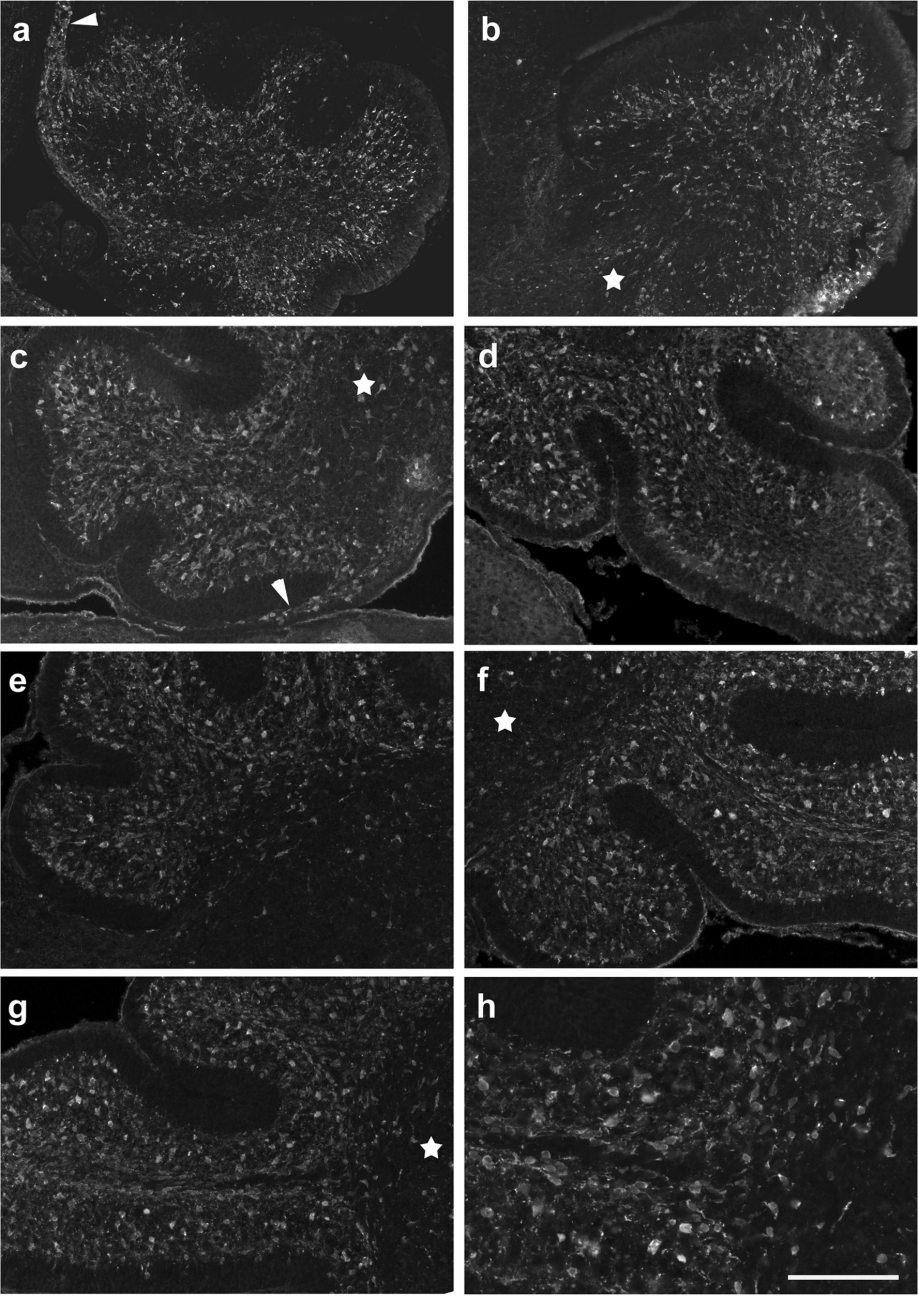
Distribution of Pax2-positive precursors of inhibitory interneurons (Pax2 cells) in the p0–p6 cerebellar anlage. **a**, **b** Sections close to the midline (**a**) and the lateral (**b**) cerebellar anlage at p0. Note the presence of Pax2 cells in the velum medullare (arrowhead in **a**) and throughout the cerebellar anlage proper, except in the external granule cell layer (EGL). The latter may be discerned due to its weak background staining. Note also that the density of Pax2 cells is lower in the region of the deep nuclei. **c**–**h** At p4, Pax2 cells are again seen in the velum medullare (arrowhead in **c**). Both at p4 (**c**, **d**) and p6 (**e**–**h**), Pax2 cells are preferentially localized in the nascent white matter, but also the nascent molecular (ML) and inner granule cell layers (IGL), both in the anterior (**c**, **e**) and the posterior (**d**, **f**) cerebellum. In the IGL, larger Pax2 cells are prominent. Pax2 cells are again excluded from the EGL, and their density in deep nuclei is rather low (star in **c**, **d**, **f**, **g**). Panels **g** and **h** show details from lobule 4. Note that positive cells are found throughout the white matter of this lobule, but preferentially localize to the margins close to the IGL. For all panels except **g** and **h**, anterior is to the left. Scale bar (in **h**) = 210 μm for panels **a**, **b**; 160 μm for panels **c**–**g**; 80 μm for panel **h**

**Fig. 2:**
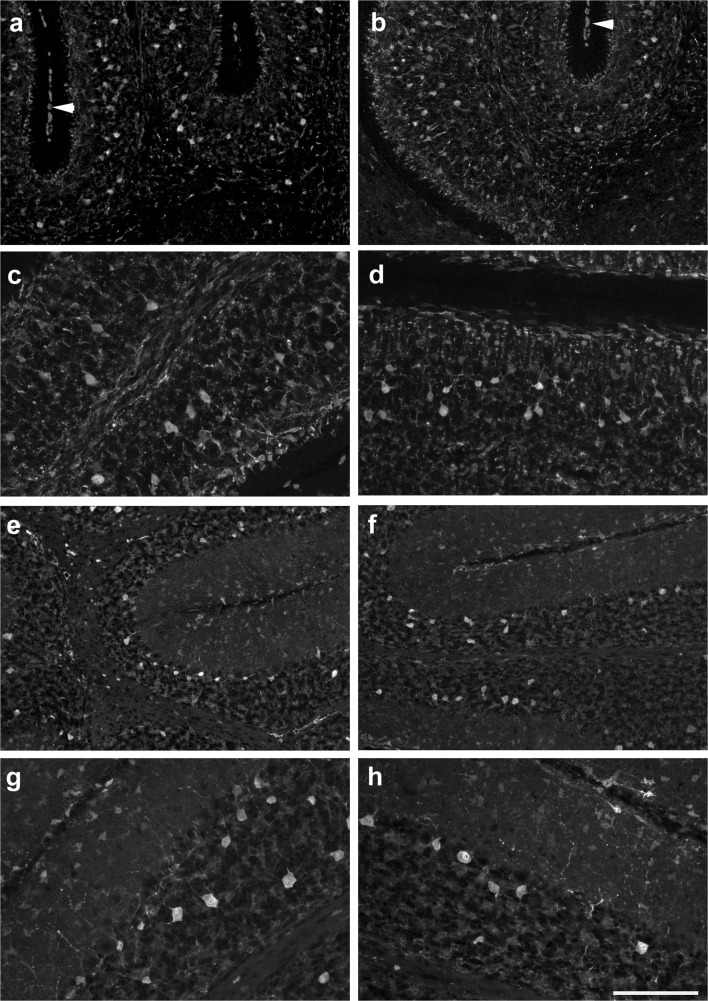
Distribution of Pax2 cells in the p8–p15 cerebellar anlage. **a**–**d** At p8, the differential arrangement of Pax2 cells in distinct layers allows to identify the latter. There still remain some positive cells in the white matter; large positive somata are restricted to the IGL, and in the ML, staining intensity is stronger close to the border of the ML and the EGL than in the deeper ML. The EGL is again devoid of Pax2 cells. Pax2 cells in the meninges (arrowheads in panels **a**, **b**) may be vascular cells (see “Discussion”). **e**–**h** In the cerebella of p15 mice, the white matter contains only a few Pax2 cells. The IGL may be easily discerned due to the presence of large-bodied positive cells. In the ML, clearly Pax2-positive cells are concentrated close to the meningeal surface. In the deeper ML, Pax2 staining is very weak. Scale bar (in **h**) = 160 μm for panels **a**, **b**, **e**, **f** and 80 μm for panels **c**, **d**, **g**, **h**

In Figs. 1 and 2, we document the distribution of Pax2 cells in the developing cerebellar anlage to facilitate comparison with the expression patterns of markers used to characterize the developing white matter (WM) described in the following paragraphs. At postnatal day 0 (p0; Fig. 1a, b), Pax2 cells appear distributed relatively evenly throughout the cerebellar anlage, with the exception of the external granule cell layer (EGL). They may also be found in the velum medullare (arrowhead in panel a). At p4 (Fig. 1c, d), the deep cerebellar nuclei (DCN) may be delineated as a region with a relatively low density of Pax2 cells (stars in panels c, d). In the nascent inner granule cell layer (IGL), many large Pax2 cells may be found. While the EGL is again devoid of Pax2 cells, many of them can be found throughout the nascent white matter (WM) and again within the velum medullare. At p6 (Fig. 1e–h), Pax2 cells in the nascent WM are even more prominent. Two days later, at p8, Pax2 cells are still found throughout the WM, although at a lower density than at p6 (Fig. 2a–d). In the IGL, large Pax2 cells are particularly prominent, and in the nascent molecular layer (ML), strongly stained Pax2 cells, with preferentially horizontally oriented somata, are found close to the border with the EGL. In the deeper ML, Pax2 expression is weaker, and there, Pax2 cell somata appear preferentially roundish (Fig. 2d). At p15, only an occasional Pax2-positive cell may still be found in the white matter. Large-bodied positive cells are prominent in the (inner) granule cell layer. In the ML, many clearly Pax2-positive cells can be seen close to the meningeal surface, while few weakly Pax2-positive cells may be discerned in the deeper regions of the ML (Fig. 2e–h).

### Maturation of Cerebellar Oligodendrocytes

CNP-1 immunostaining was used to detect early maturing oligodendrocytes and oligodendrocyte precursor cells. It is known to be expressed during early oligodendrocyte development, even before other myelin proteins such as MBP, and its expression persists until adult ages [19–22]. In more mature oligodendrocytes, the immunosignal of CNP-1 is typically distributed rather homogeneously [20] and thus allows to outline these cells. MBP is yet another differentiation marker for oligodendrocytes which has been reported to be expressed 2–3 days after CNP-1 in rodent cerebellar oligodendrocytes [23, 24]. We used both of these markers to map the maturation of cerebellar oligodendroglia. As both markers are myelin-associated, we expected a staining pattern as typical for membranes.

#### Invasion and Settling of CNP-1-Positive Oligodendrocytes in the Cerebellar Anlage

**Fig. 3:**
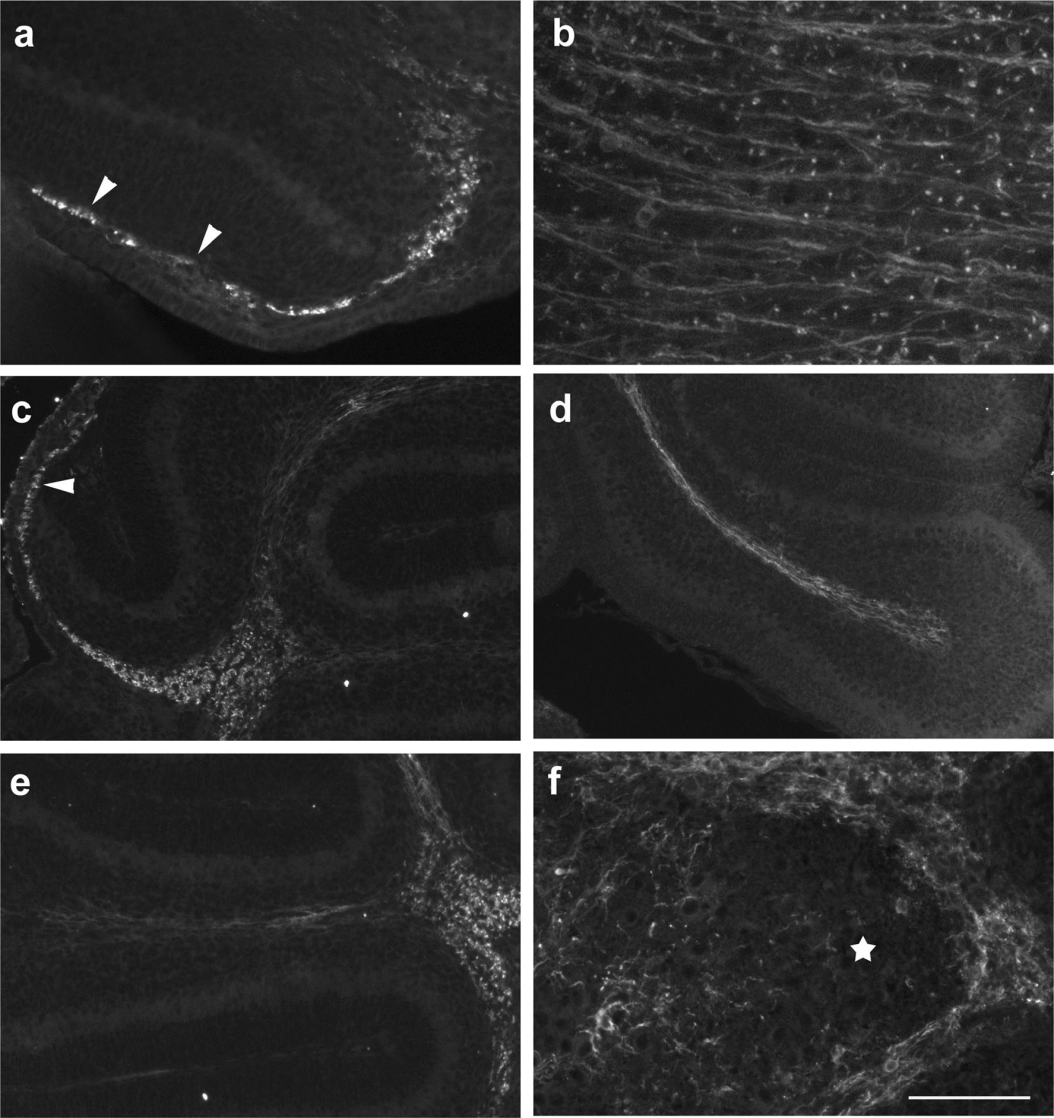
CNP-1 immunoreactivity in the early postnatal mouse cerebellum (p0–p6). **a** CNP-1-positive cells in the p4 cerebellar anlage are roundish and may be found in the velum medullare (arrowheads in **a**) and the anteriormost parts of the cerebellar anlage. Sagittal section close to the midline; anterior is to the left. **b** In the brainstem, CNP-1-positive structures have a fibrous morphology at this age. **c**, **d** CNP-1-positive cells at p6. Immunoreactive cells can again be detected in the velum medullare (arrowhead in **c**). They can now also be seen in the nascent central WM and the nascent WM extending into the anterior and posterior lobules (**d**; lobule 8). Sagittal sections close to the midline. Posterior is to the right. **e**, **f** In median lobules (**e** shows lobule 4; superior is to the left) CNP-1 expression is lower, but appears already fibrous, whereas staining in the central deep cerebellar mass (to the right of the panel) appears still granular at p6. Panel **f** displays a region from around deep cerebellar nuclei (marked by a star). These are surrounded by a rather dense fibrous immunopositive network. Some stained fiber-like structures can also be discerned within deep nuclei. Scale bar (in **f**) = 80 μm for panels **a**, **b**, **f** and 160 μm for panels **c**, **d**, **e**

At p0, we could not detect any CNP-1 immunopositive structures in the cerebellar anlage, while numerous CNP-1-positive cells were visible in the brain stem. In contrast, at p4, CNP-1 immunoreactivity could be seen in the velum medullare and rostral central mass of the cerebellum (Fig. 3a). It appeared granular, and individual cells could not really be delineated. Larger immunopositive granula were typically seen surrounding cell nuclei. In contrast, the obviously more mature CNP-1-positive cells in the brainstem showed an extended, fibrous morphology (Fig. 3b).

At p6, CNP-1-positive structures could be detected throughout the central WM of the cerebellum (Fig. 3c). At this age, CNP-1 immunoreactivity was also visible in the in the prospective WM of the posterior lobules (Fig. 3d), and of the first two anterior lobules (Fig. 3c). Intralobular CNP-1-positive structures could be detected up to the tip of the lobules, but not yet in the IGL. In contrast, at best very few CNP-1-positive structures could be seen in the medial lobules, primarily at their basis (Fig. 3e, f). While CNP-1-immunoreactivity appeared mostly granular, and individual cells could not reliably be delineated in the central parts of the cerebellum, immunostained structures in the nascent lobular WM of anterior and posterior lobules were elongated and reminiscent of long fibrous processes (Fig. 3c, e). Around the DCN, there was a dense, CNP-1-positive fibrous network. Somata of individual oligodendrocytes were hard to discern. CNP-1-positive structures were also visible within DCN (Fig. 3f).

**Fig. 4:**
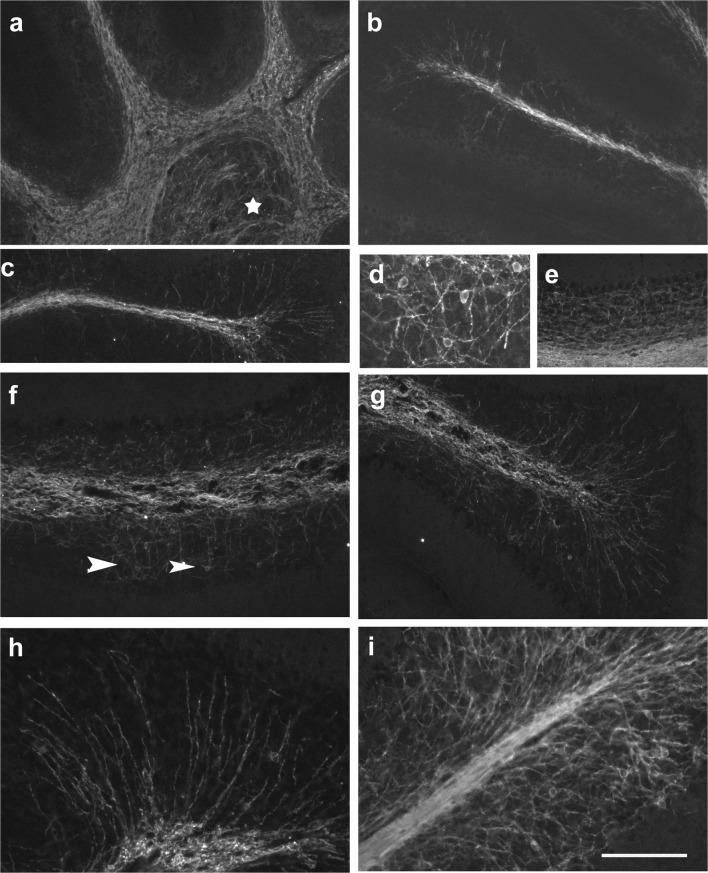
CNP-1 immunoreactivity in the p8–p15 cerebellum. **a**–**c** CNP-1-positive structures in the p8 cerebellum. Note dense staining throughout the white matter and the fibrous morphology of all CNP-1-positive cells. A few CNP-1-positive cells are also present within the deep cerebellar nuclei (star in **a**). CNP-1-positive fibers reach the tips of all lobuli (shown for lobule 4 in panel **b** and lobule 8 in panel **c**) and can also be seen penetrating the IGL (**b**, **c**). No CNP-1-positive somata can be detected within the IGL (**c**). **d**, **f**, **g**, **h** CNP-1-positive oligodendrocytes in the p15 cerebellum. Note CNP-1-marked fibers transversing the IGL (**f**, **g**), where also stained somata are detectable (**d**; arrowheads in **f**). **i** At p28 and in the adult (**e**), a dense complement of CNP-1-positive fibers is present throughout the granule cell layer and the white matter. Scale bar (in **i**) = 160 μm for panels **a**, **b**, **c**, **e**, **f**, **g** and 80 μm for panels **d**, **h**, **i**

Two days later, at p8, CNP-1-positive oligodendrocytes were found distributed throughout the developing WM (Fig. 4a), up to the tips of posterior and anterior lobules (Fig. 4b, c). They mostly displayed a fibrous morphology, and in contrast to p6, granular CNP-1 staining was much reduced in the central parts of the cerebellum. Further, a few CNP-1-positive fibers could now also be detected within the IGL, where they were essentially restricted to the region abutting the WM (Fig. 4b, c). These were somewhat irregularly distributed and recalled the strands of a bottlebrush. CNP-1-positive somata could not be detected within the IGL. As at p6, CNP-1-positive oligodendrocytes were also found in the DCN (marked by a star in Fig. 4a). Throughout the nascent WM, the density of CNP-1-marked fibers had increased as compared with p6, and fibrous processes appeared much elongated. CNP-1-positive somata were preferentially discernible in areas where the density of oligodendrocytes (and their immunostained fibrous processes) was rather low.

By p15, an even denser network of CNP-1-positive oligodendrocytes throughout all of the cerebellum and up to the Purkinje cell layer (PCL) had developed. At the tips of the lobular WM, CNP-1-positive fibers entering the IGL branched radially into all directions (Fig. 4g). They spanned the entire IGL layer, where now also CNP-1-positive somata could be found (Fig. 4d, f). Typically CNP-1-positive fibrous structures were oriented more or less perpendicularly to the border of the WM and IGL (Fig. 4f, g). Moreover, we detected CNP-1-positive somata in the outer part of the IGL, the extensions of which formed CNP-1-positive fibers orientated parallel to the border between the IGL and PCL (Fig. 4h).

By p28 (Fig. 4i) and in the adult (Fig. 4e), we found an even denser network of CNP-1-positive fibers throughout the WM and the granular cell layer, again up to the PCL, and also within DCN.

At no age could we detect CNP-1-positive structures in the (nascent) ML, nor in the EGL.

#### Appearance of MBP-Positive Oligodendrocytes in the Cerebellar Anlage

**Fig. 5:**
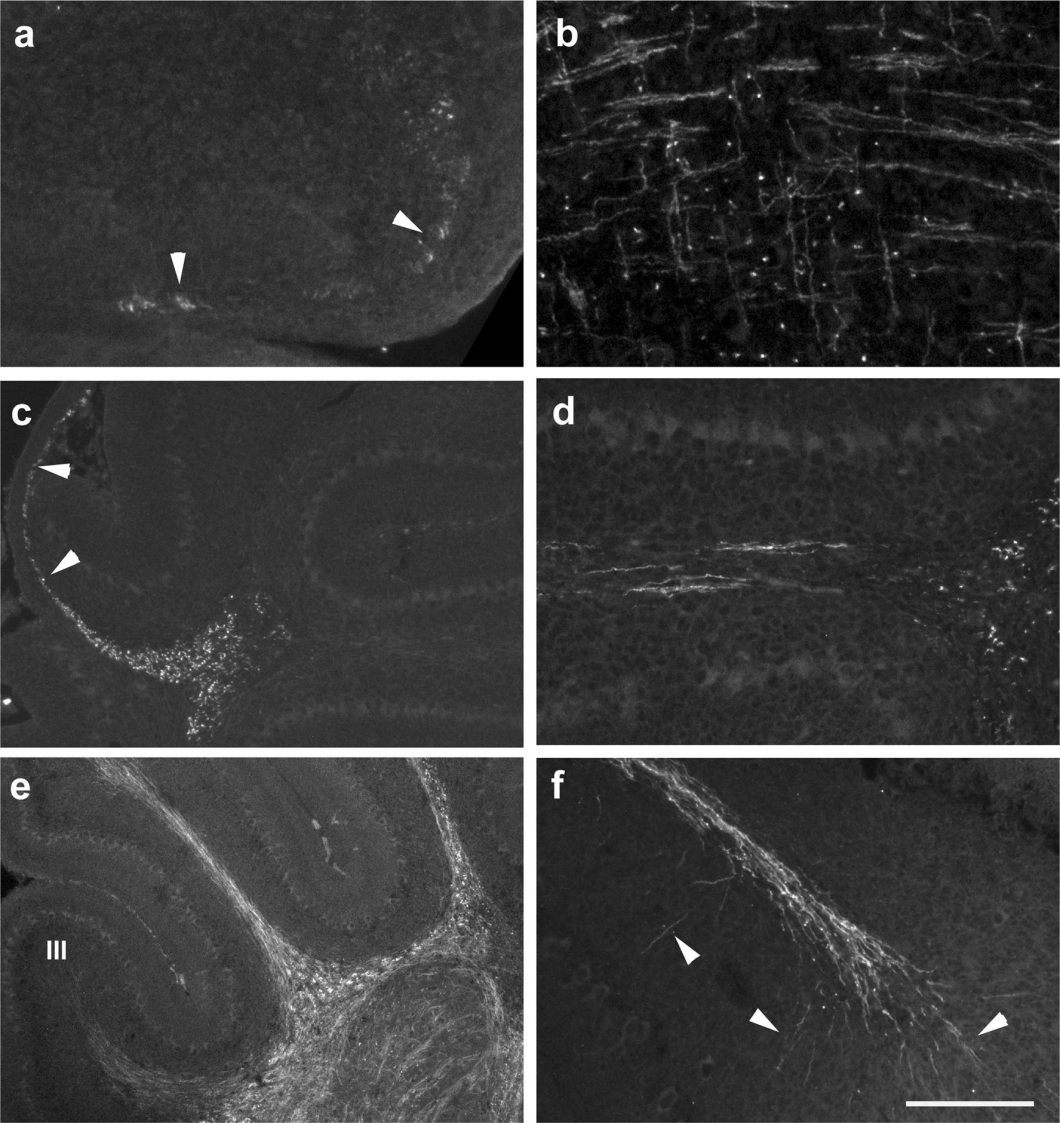
MBP immunoreactivity in the early postnatal mouse cerebellum (p4–p8). **a**, **b** MBP-positive cells in the p4 cerebellar anlage are roundish and restricted to the velum medullare (arrowheads in **a**). For comparison, MBP-positive structures in the p0 brainstem have a fibrous appearance (**b**). **c**, **d** At p6, roundish MBP-positive cells are present in the velum medullare (arrowheads in **c**), the central cerebellar WM, and at the bases of individual lobules (**c**). Within the nascent lobular WM, a few fibrous MBP-positive structures may be discerned (**d**; basal is to the right; apical to the left.). **e**, **f** MBP-positive structures within the WM of the p8 cerebellum are intensely stained, and some weaker staining is also present within the DCN (**e**). All MBP-positive structures show a fibrous morphology. While within the anterior lobules (**e**; lobule III is marked), MBP-positive structures do not reach the tips of the lobular WM, they may be found up to the tips of the posterior lobules, where they branch into few thin short strains that enter the IGL in nearly rectangular angles (arrows in **f**). Scale bar (in **f**) = 80 μm for panels **a**, **b**, **d**; 160 μm for panels **c**, **f**; and 210 μm for panel **e**

At p0, both the cerebellum and the adjacent brainstem were negative for MBP. At p4, only a very few dot-like MBP-positive oligodendrocytes could be detected in the velum medullare (Fig. 5a), but no MBP-positive cells were present within the cerebellum proper. In contrast, in the p4 brainstem, strongly MBP-positive cells stood out as fiber like structures (Fig. 5b).

By p6, many MBP-positive oligodendrocytes could be seen in the velum medullare and the central cerebellar WM (Fig. 5c). MBP-positive cells were also found in the transitory region joining the central WM with that of individual lobules. In the nascent lobular WM, only a few MBP-positive cells were present, preferentially in the middle, i.e., about equidistant from the abutting IGLs (Fig. 5d). The distal parts of the nascent lobular WM were still MBP-negative. While MBP-expressing cells in the central WM and the velum medullare were preferentially roundish, those within lobules had a fibrous morphology (Fig. 5d).

In p8 mice, we found a dense population of MBP-positive oligodendrocytes throughout the entire cerebellar WM (Fig. 5e). In the anterior lobules MBP-expressing cells had not yet reached the tips of the individual lobules, and the IGL was as well still devoid of MBP-positive structures (Fig. 5e). Posterior, MBP-positive, fibrous structures could be detected up to the tips of the individual lobules, where they branched into thin and short strains (Fig. 5f). There, a few MBP-positive oligodendrocytes were also found in the inner part of the IGL. These were orientated at nearly right angles relative to the border of the IGL and the WM (Fig. 5f). MBP-positive oligodendrocytes could also be found within the DCN (Fig. 5e). By now, all MBP-positive cells in the cerebellum, including those in the IGL, had a fibrous structure.

**Fig. 6:**
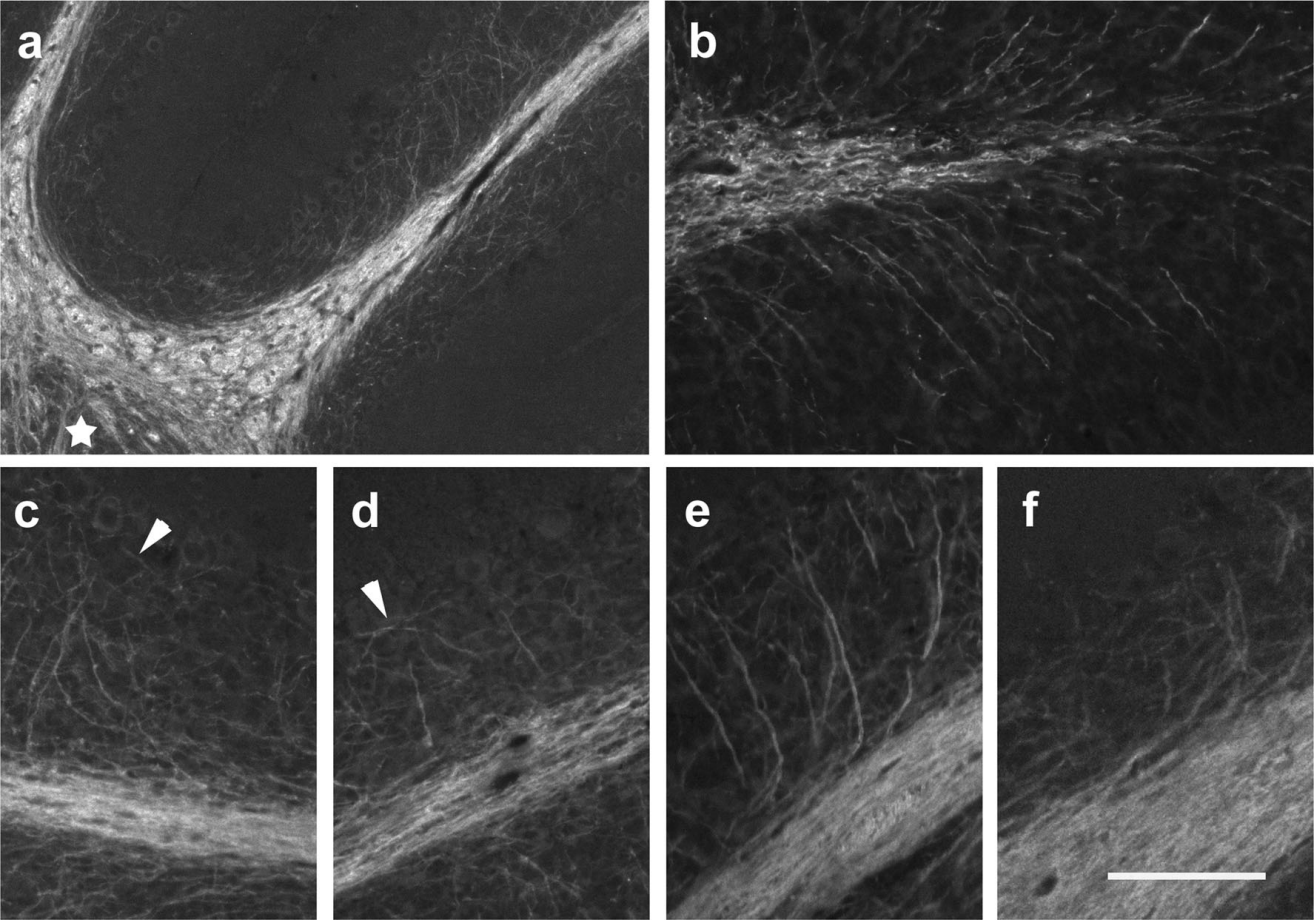
MBP immunoreactivity in the p15 and p28 cerebellum. **a**–**d** The p15 cerebellum is characterized by intensive immunoreactivity for MBP throughout the WM. The density of MBP-positive fibers also increased within the DCN (star in **a**) and the IGL of all lobules. In the proximal parts of the lobules, MBP-positive fibers are found almost up to the PCL (**a**, **c**). Distally, MBP-positive fibers do not yet reach the PCL (**b**, **d**). At the tips of the lobular WM, MBP-positive fibers branch radially in all directions (**b**). Arrowheads in **c** and **d** point out MBP-positive fibers running more or less parallel to the PCL. **e**, **f** In the p28 cerebellum, many MBP-positive fibers ascend through the IGL up to the PCL. Whereas they emanate at flat angles from the white matter in proximal parts of the lobules (**e**), more distally, they are oriented nearly rectangular to the white matter (**f**). Scale bar (in **f**) = 160 μm for panel **a** and 80 μm for panels **b**–**f**

By p15, the density of MBP-positive oligodendrocytes had increased throughout the cerebellum (Fig. 6a). A network of such cells had formed within the IGL, where it appeared denser in its lower than in its upper part (Fig. 6b–d). Moreover, within the proximal parts of the lobules, MBP-positive structures reached (almost) up to the PCL (Fig. 6c), while in distal parts they were preferentially found within the inner third of the granule cell layer (Fig. 6d). MBP-positive cells within the granule cell layer located close to the WM were often arranged in strand-like groups that were more or less perpendicularly arranged relative to the border of the granule cell layer and the WM (Fig. 6b–d). Closer to the PCL, the orientation of MBP-positive oligodendrocytes was more variable.

At p28 and in adult animals, MBP-positive oligodendrocytes appeared even denser, especially in the granule cell layer (Fig. 6e, f). MBP-positive fibers in proximal parts of the lobules often emanated from the WM in flat angles (Fig. 6e); more distally, they were orientated more or less perpendicular to the central WM in distal parts of the lobules (Fig. 6f).

**Fig. 7:**
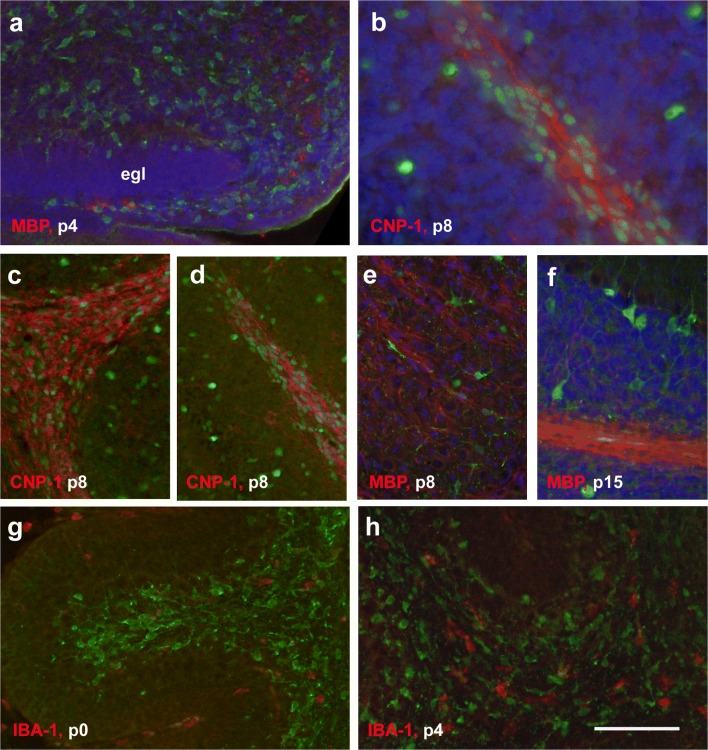
Costaining for Pax2-GFP and markers for oligodendrocytes or microglia. **a** At p4, only a few MBP-positive oligodendrocytes (red) can be detected. These are restricted to the velum medullare, whereas Pax2 cells (green) are spread rather homogeneously in velum medullare and the nascent cerebellum, with the exception of the EGL. The EGL of lobule 1 is marked. **b**–**d** High power view (**b**) of the lobular WM of lobule 4/5 at p8. CNP-1-positive oligodendrocytes (red) are restricted to the WM. In contrast, Pax2 cells (green) are found both in the cerebellar cortex and the WM. In the WM, Pax2 cells and oligodendrocytes are mixed rather homogenously and show no obvious association, as documented for the basis (**c**) and tip (**d**) of lobule 4/5. **e** DCN at p8. Pax2 cells and MBP-positive oligodendrocytes (red) appear distributed throughout deep nuclei rather evenly and reveal no special association. **f** Lobule 4/5 at p15. Pax2 cells have now mostly migrated into the cerebellar cortex, while MBP-positive oligodendrocytes are restricted to the WM and IGL. **g**, **h** At p0 (**g**), both Iba-1positive microglia (red) and Pax2 cells (green) can be detected in the cerebellar anlage. They intermingle in the prospective WM, but no spatial association can be observed. The same is true at p4 (**h**). In panels **a**, **b**, **e** and **f** nuclei are counterstained in with Hoechst (blue). Scale bar (in panel **h**) = 80 μm for panels **a** and **c**–**h** and 40 μm for panel **b**

The distribution of CNP-1- and MBP-defined oligodendroglia relative to that of Pax2-defined inhibitory interneurons and their precursors is documented in Fig. 7a–f. While, as detailed above, at p4, oligodendroglia could only be detected in the velum medullare, Pax2-positive interneuronal progenitors were spread rather homogeneously throughout the cerebellar anlage and the velum medullare (Fig. 7a). By p8, when oligodendroglial structures were found up to the tips of the lobular WM and also in the lower regions of the IGL (cf above), Pax2 cells were found interspersed with oligodendrocytes within the cerebellar WM, but also throughout the cortex still devoid of MBP- or CNP-1-positive oligodendrocytes (Fig. 7b–e). The large, strongly Pax2-positive interneurons in the IGL of the p15 cerebellum were not associated with structures marked by CNP-1 or MBP (Fig. 7f). Thus, we could not detect any systematic spatial relation between oligodendrocytes and Pax2 cells at any age studied.

### GFAP-Defined Astroglial Maturation

To visualize the development and distribution of differentiated astrocytes, including Bergmann glia, we used immunostaining for GFAP [25].

**Fig. 8:**
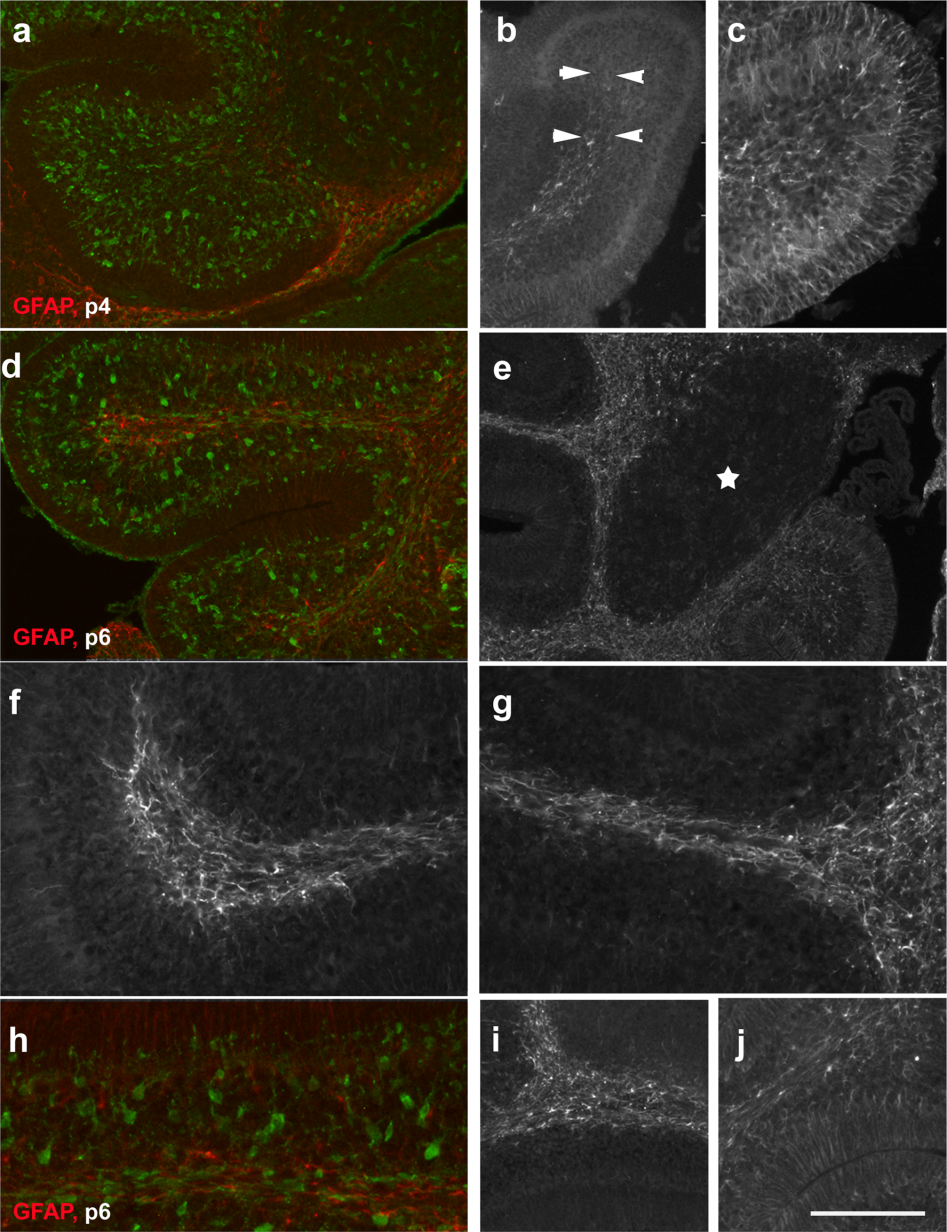
Expression of GFAP in the early postnatal cerebellum (p4–p6). **a**–**c** GFAP-immunoreactivity can first be seen on p4 in the velum medullare and adjoining region of the cerebellar anlage (**a**, red). A few GFAP-positive cells can also be detected in the central WM surrounding the DCN (star) and at the bases of all lobules. Within lobules, the prospective WM is essentially devoid of GFAP-positive cells (**a**, red) with the exception of the posterior lobules (**b**, **c**), where GFAP-positive cells are found up to the tips of the lobular WM (arrows in **b**) and in the cerebellar cortex (**c**). In panel **a**, Pax2 cells are labeled green. Note that the DCN are immunonegative for GFAP. **d**, **e** At p6, the density of GFAP-positive cells (red in **d**) has increased and they can be detected in both the central and lobular WM up to the tips of the individual lobules. The DCN (**e**, star) remain immunonegative for GFAP. In panel **d**, Pax2 cells are labeled green. **f**–**j** Anterior-posterior differences of GFAP immunoreactivity at p6. In the medial lobules (**f**, tip of lobule 4; **g**, basis of lobule, 4; **i**, lobule 6/7), only a few Bergmann glia are immunopositive for GFAP, and only a few GFAP-positive cells can be detected in the IGL. In the posterior lobules (**j**), Bergmann glia and astrocytes in the IGL show stronger staining. Note, however, that the density of GFAP-positive cells in the lobular white matter seems to be higher in medial than in posterior lobules. Panel **h** shows part of the anterior lobule 3 stained for GFAP (red) and Pax2 (green). Scale bar (in **j**) = 160 μm for panels **a**, **b**, **d**, **i**, **j**; 80 μm for panels **c**, **f**, **g**; and 210 μm for panel **e**

At p0, we could not detect any GFAP-positive cells in the cerebellar anlage. In contrast, at p4, GFAP-positive cells were found throughout the velum medullare and in the transitory region joining the velum to the cerebellum proper. GFAP-positive cells were also visible within the central cerebellar WM and the bases of all lobuli, although at lower density (Fig. 8a). The distal parts of the nascent lobular WM were devoid of GFAP-positive structures (Fig. 8a), with the notable exception of the posterior lobules, where GFAP-positive cells could be seen up to the distal WM (arrows in Fig. 8b). Indeed, in posterior lobules, a few GFAP-positive cells could already be seen in the nascent IGL, and also the nascent ML (Fig. 8c). In contrast, in the anterior lobules, the IGL, just like the PCL and the ML, were GFAP-negative at p4 (Fig. 8a). The DCN were immunonegative for GFAP (Fig. 8a).

In p6 mice, we found GFAP-positive astrocytes in all of the prospective cerebellar WM, where they reached the tips of all lobuli (Fig. 8d, e). Staining of the cortex, including the ML, was weaker and showed clear antero-posterior differences. Whereas in posterior lobes (Fig. 8j) individual Bergmann glial fibers became discernible, staining of the ML and IGL in the central lobules was much weaker and diffuse (Fig. 8f, g, i, j). The anterior lobules showed an intermediate level of cortical staining (Fig. 8h). The DCN were negative for GFAP (Fig. 8e).

**Fig. 9:**
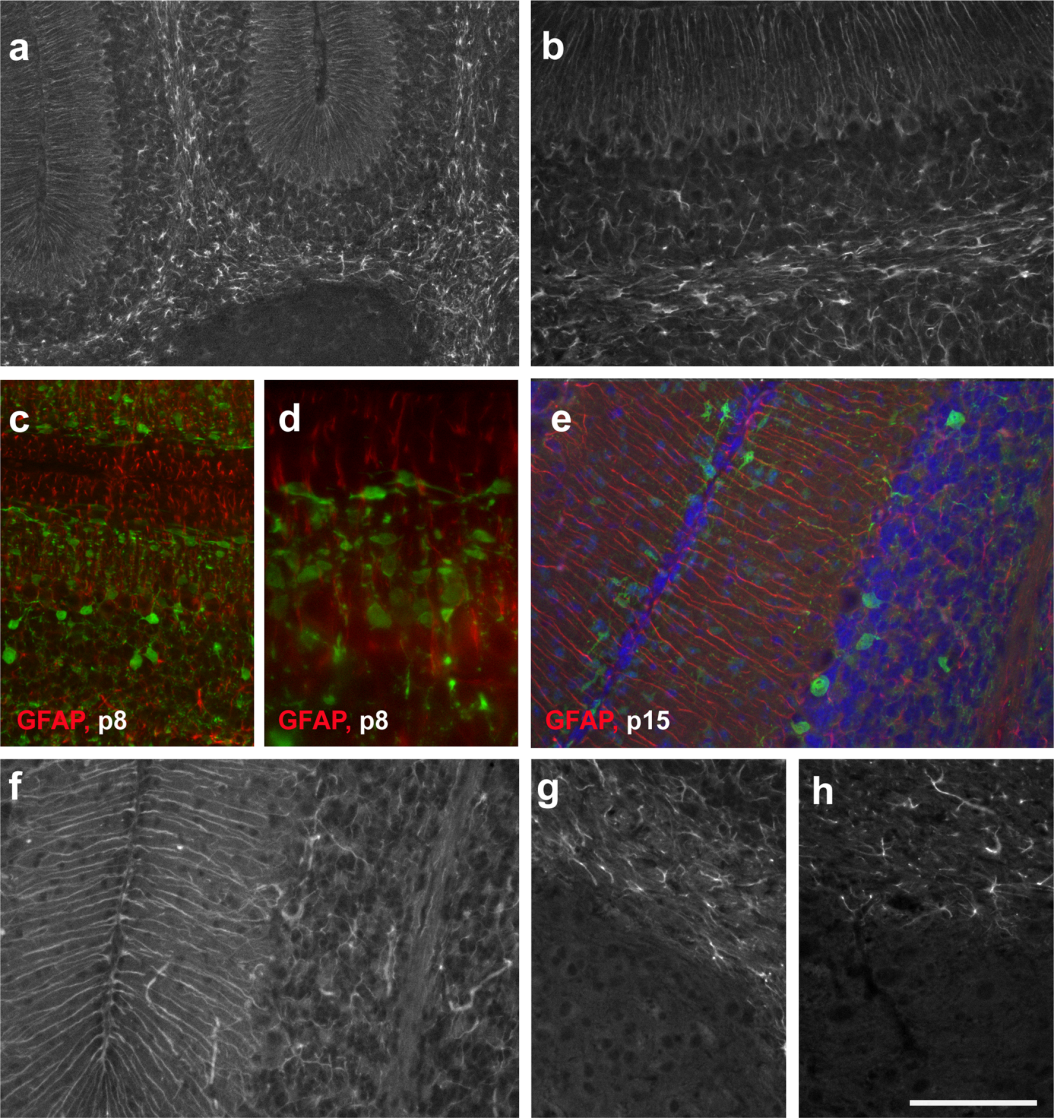
Expression of GFAP in p8–p28 cerebellum. **a**–**d** At p8, GFAP-positive cells are found in the central and lobular WM, the IGL and as Bergman glia in the PCL reaching up to the meninges. DCN are GFAP negative. The higher power view shown in **b** documents the layer-specific orientation of GFAP-positive processes. In the ML (**c**, **d**; GFAP shown in red; Pax2 in green), GFAP-stained Bergmann glial processes are cut preferentially longitudinally, whereas in the EGL, they are cut transversally. Note that processes of Pax2 cells underneath the EGL run essentially perpendicular to those of Bergman glia (**c**, **d**). **e**–**g** GFAP-positive structures in the p15 cerebellar cortex (**e**; GFAP = red; Pax2 = green; nuclei = blue), cortex and adjacent white matter (**f**), and in the central white matter and deep nuclei (**g**). Note that the latter are again immunonegative for GFAP. Note also that strongly Pax2-positive cells are restricted to the IGL and a small strip right underneath the remnant of the EGL at this age. Interneurons in the deeper ML are only very faintly Pax2-positive. **h** In the p28 cerebellum, the DCN remain immunonegative for GFAP. Scale bar (in panel **h**) = 160 μm for panel **a**, and 80 μm for panels **b**–**g**

By p8, the density of GFAP-positive astrocytes had again increased throughout the cerebellum (Fig. 9a). Well delineated GFAP-positive cells were now visible within the IGL of all lobules. Also, Bergmann glia fibers in the ML and penetrating the EGL were now also more intensely stained and readily discernible (examples are shown in Fig. 9a–d). Again, the DCN stood out as a region devoid of GFAP-positive astrocytes (Fig. 9a). GFAP-positive glial processes were already arranged in the layer specific orientation typical for adult glia, e.g., [2, p. 69, Fig 50]: within the WM, astrocytic processes were preferentially oriented parallel to the PCL; in the IGL, they had no preferential orientation, giving astrocytes their typical star-shared appearance; and Bergmann glia in the ML and EGL were arranged perpendicular to the PCL (Fig. 9b, c, d). Of note, the exact appearance of GFAP-positive Bergmann glial fibers also reflected the distinct cellular compositions of the layers they traversed. In the ML, some could be seen to branch and staining was somewhat more diffuse, whereas in the EGL, fiber processes appeared straight and rather unbranched (Fig. 9b).

By p15, GFAP-positive astrocytes in the WM showed an even stronger orientation parallel to fiber tracts and often appeared as slender, bipolar, rather than star-shaped cells Fig. 9e, f). The density of Bergmann glia fibers in the ML (Fig. 9e, f) had again increased, and with the dissolution of the EGL, their appearance up to the meninges now was rather uniform. As at younger ages, GFAP-positive astrocytes could not be detected within the DCN (Fig. 9g). The distribution and appearance of GFAP-positive astrocytes at p28 and in adult animals was very much the same as at p15. Notably, DCN were devoid of GFAP-positive structures also at these ages (Fig. 9h).

Comparison of sections stained for Pax2 (Figs. 1 and 2) with those for GFAP (Figs. 8 and 9) as well as inspection of selected sections labeled for both Pax2 and GFAP (Fig. 8a, d, h; Fig. 9c–e) indicates that Pax2 cells settling the cerebellar cortex early on do not go through areas where they might contact, or get near, GFAP-positive cells. In contrast, those that immigrate into the cerebellar cortex around and beyond p4 have to transit the nascent WM at a time when it is already densely populated by GFAP-positive astrocytes. Double labeling for GFAP and Pax2 also highlights that while Bergman glial processes penetrate the EGL, Pax2 cells are excluded from this layer (Fig. 9c–e).

### Iba-1-Positive Microglia in the Cerebellar Anlage

**Fig. 10:**
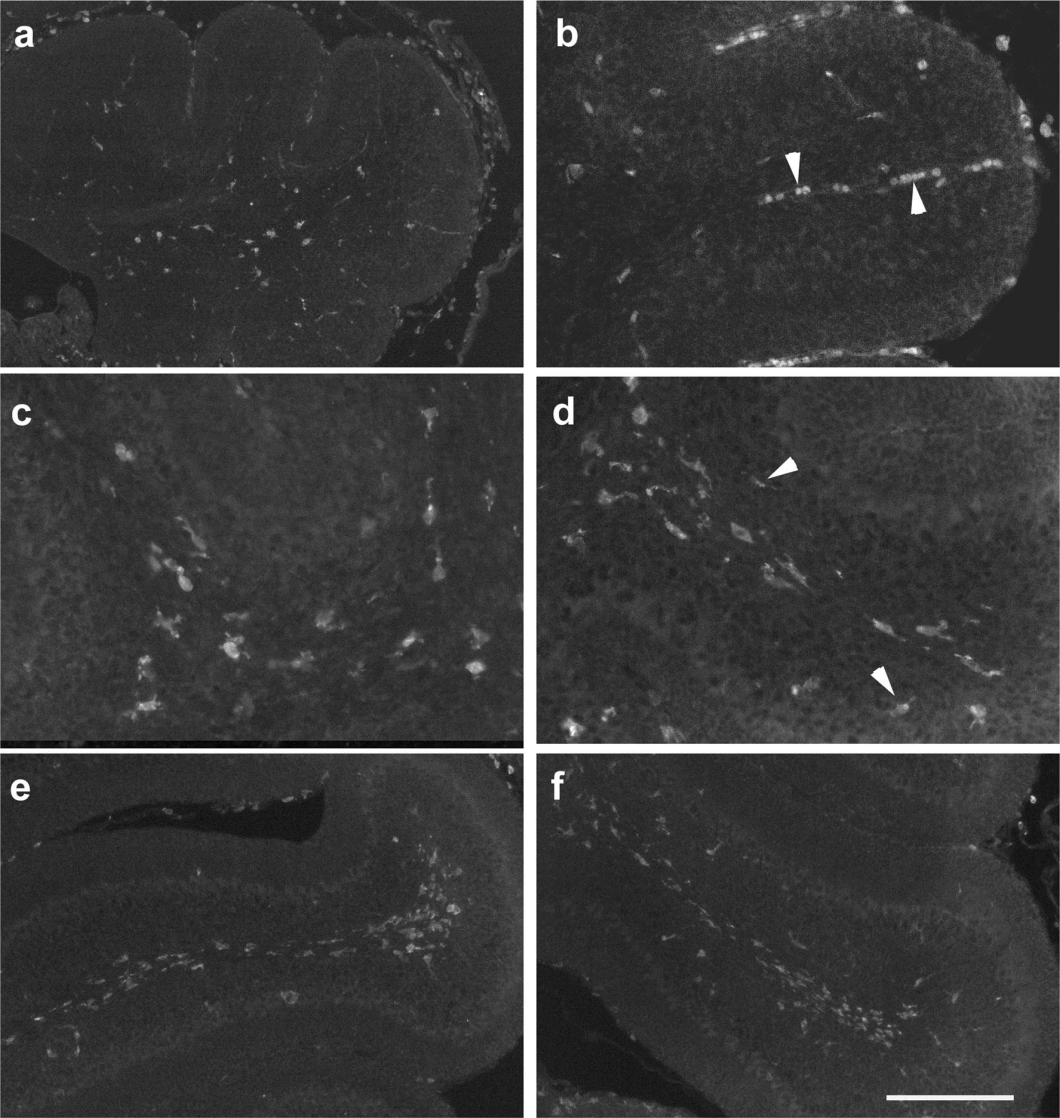
Iba-1 expression in the cerebellum from p0 to p6. **a**, **b** Iba-1-positive cells in a midsagittal section of the cerebellum of a newborn (p0) mouse (**a**). Cells appear roundish and are often associated with blood vessels (**b**, arrowheads) and the meninges. **c**, **d** At p4, Iba-1-positive cells are spread throughout the cerebellar WM up to the tips of individual lobules (**d**). In the IGL, only a very few Iba-1-positive cells can be detected (arrowheads in **d**). Iba-1-positive cells have roundish somata with short, thick processes which are stronger branched than at p0 (**c**). **e**, **f** At p6, numerous Iba-1-positive cells are found throughout the WM (**e**, lobule 4; **f**, lobule 8). A few Iba-1-positive cells can also be detected in the IGL up to the PCL. While the cells located in the WM display a thin and straight morphology, cells located in the IGL appear more roundish (**f**). In the tips of the WM of the anterior lobules, roundish Iba-1-positive cells accumulate (**e**, lobule 4). Scale bar (in **f**) = 210 μm for panel **a**; 80 μm for panels **b**–**d**; and 160 μm for panels **e**, **f**

Iba-1 staining was used to identify and localize microglia [26] in the developing cerebellum. At p0, Iba-1-stained microglial cells were visible throughout the nascent WM of the cerebellum, preferentially in its central parts (Fig. 10a). Many Iba-1-positive cells were associated with larger intracerebellar blood vessels and meninges (Fig. 10b, blood vessel marked by arrows). Within the velum medullare, the density of Iba-1-positive cells was comparable to that in the cerebellum proper (not shown). Iba-1-stained cells were roundish or polymorph, with only few processes (Fig. 10a, b).

At p4, the restriction of Iba-1-stained microglia to the nascent WM was even more obvious than at p0 (Fig. 10c, d). They appeared distributed more or less evenly up to the tips of the lobular WM (Fig. 10d) and around the DCN. A few Iba-1-positive cells could also be detected in the lower IGL (Fig. 10c, d). The remainder of the cerebellar cortex and the DCN were devoid of Iba-1-positive cells, except for those associated with larger penetrating blood vessels (Fig. 10c). Again, many Iba-1-positive cells were found in the meninges. At this age, Iba-1-stained cells had roundish somata from which a few short processes emanated. These were more branched than at p0 (Fig. 10c).

At p6, numbers of Iba-1-stained microglia had increased compared with p4 (Fig. 10e, f). Again, most Iba-1-positive microglia could be found in the central and lobular WM (Fig. 10e, f), but a few were also detectable in the IGL and PCL (Fig. 10f). While Iba-1-stained microglia within the central and lobular WM now were thin and longish, those that had accumulated in the lobular tips were rather roundish (Fig. 10e). This morphological difference was more prominent in lobule 4 (Fig. 10e) than in posterior lobules (Fig. 10f). Iba-1-positive cells in the IGL were also roundish (Fig. 10e).

**Fig. 11:**
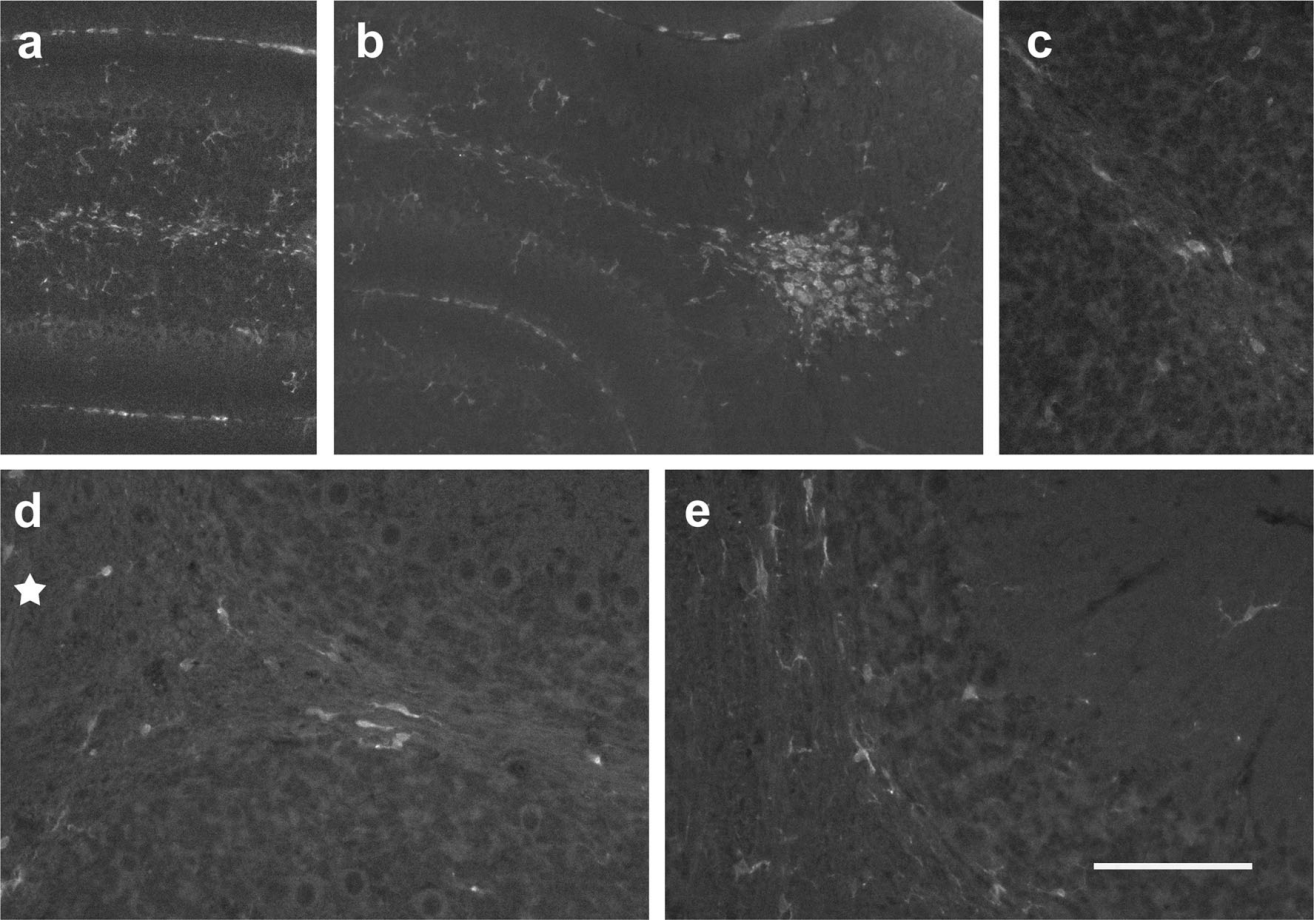
Iba-1 expression in the cerebellum from p8 to p28. **a**, **b** Exemplary view of Iba-1-positive cells in the p8 cerebellum taken from lobule 4. In the lobular and central WM, the density of Iba-1 cells is higher than in the IGL. A few Iba-1-positive cells can also be seen in the nascent ML. The meninges are also intensely Iba-1-immunoreactive. Note morphological variability of immunoreactive cells in the cortex and nascent WM. At the tips of the lobular white matter (**b**, to the right), aggregations of larger, roundish Iba-1-positive somata prevail. **c**–**e** In the p15 (**c**, **d**) and p28 (**e**) cerebellum, Iba-1-positive cells are mainly found in the WM, with a few cells also in the IGL, the ML and in the DCN (star in **d**). At p15, they appear mostly as small, longish cells that became increasingly ramified at p28 (compare **d** and **e**). The overall density of Iba-1 cells is lower than at p8. Scale bar (in **e**) = 160 μm for panels **a**, **b** and 80 μm for panels **c**–**e**

At p8, Iba-1-positive cells were found at a rather high density throughout the WM (and within the meninges), and at a clearly lower density within the IGL. A few Iba-1-stained cells were now also found in the nascent ML, up to the border between the ML and the EGL (Fig. 11a). The latter appeared essentially devoid of microglial cells. Iba-1-stained microglia were also found within the DCN (not shown).

The appearance of Iba-1-positive cells differed regionally. Within the IGL, PCL, ML, and in the DCN, these cells had mostly small, roundish somata and short processes (Fig. 11a). At the tips of the lobular WM, Iba-1-positive cells had roundish cell bodies with no discernible processes found, arranged as large accumulations (Fig. 11b). In contrast, within the central and proximal lobular WM (Fig. 11a), Iba-1 cells had rather ramified processes and their cell bodies were typically not well demarcated. Together, the Iba-1 staining thus resulted in a “flower bouquet”–like appearance of the lobular WM (Fig. 11b, from lobule 4). In the posterior lobules, this distribution was not as distinctive as in the anterior lobules (not shown).

At p15, the density of Iba-1-marked microglia had much decreased compared with p8 (Fig. 11c, d). Small, longish Iba-1-positive microglial cells were detected mainly in the WM (Fig. 11c, d), and few cells were visible in the IGL and ML. A few Iba-1-marked microglia were also found within the DCN (Fig. 11d).

At p28, the density of Iba-1-positive microglia appeared again lower than at p15. Microglial cells were found in the DCN, WM, IGL, and ML (the latter three are shown in Fig. 11e). Compared with previous ages, their processes could be better discerned, and many Iba-1-positive cells showed a stellate morphology with short, thin extensions (Fig. 11e). In adult cerebella, only an occasional Iba-1-positive cell could be detected in the cerebellum (not shown). They occurred in all cortical layers, the WM and the DCN, and typically had roundish somata and a few short processes. Given the scarcity of these cells, regional differences could not be ascertained.

To facilitate the comparison between the distribution of Iba1-positive cells and Pax2 cells, a selection of double stained sections is shown in Fig. 7g, h). At no age, a direct association between Iba-1 microglia and Pax2 cells could be observed.

### Distribution of Phosphohistone H3–Defined Mitotic Cells in the Nascent Cerebellum

**Fig. 12:**
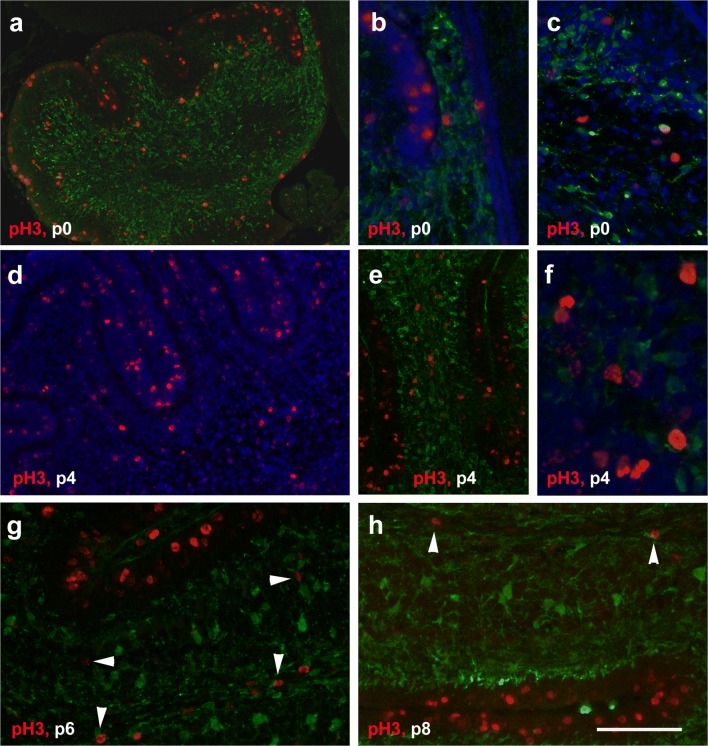
Phosphohistone H3 p0–p8. **a**–**c** In the newborn cerebellum, cells marked by pH3 (red) are detectable throughout the cerebellum including the velum medullare (**b**), with the highest density in the EGL (**a**). Pax2 cells are marked in green. Panel **c** shows one of the very few Pax2 cells also being positive for pH3 seen at any age studied. **d**–**f** At p4, pH3-positive cells are found throughout the cerebellum, but again preferentially in the EGL (**d**, **e**). The WM also comprises a higher density of pH3 expressing cells than the IGL, the ML or the DCN. pH3-positive cells within the WM show variable staining intensity and patterns (**f**). **g**, **h** At p6 (**g**) and again at p8 (**h**), numerous pH3-positive cells remain in the EGL, but such cells are also found in the (prospective) WM (vertical arrowheads in **g**, **h**). A few pH3-positive cells can also be seen in the IGL (horizontal arrowheads in **g**). In panels **b**–**d** and **f**, cell nuclei are counterstained with Hoechst (blue). Scale bar (in **h**) = 210 μm for panel **a**; 80 μm for panels **b**, **c**, **g**, **h**; 160 μm for panels **d**, **e**; and 40 μm for panel **f**

To detect dividing cells, we used staining for phosphohistone H3 (pH3), a mitotic marker. At p0, mitotically active cells marked by pH3 were detectable throughout the cerebellum (Fig. 12a), including the nascent WM. pH3-positive cells were also detectable within the velum medullare superius (Fig. 12b). With a rare exception (Fig. 12c), Pax2 cells were negative for pH3.

At p4, cells marked by pH3 were again visible throughout the cerebellum (Fig. 12d), and also the velum medullare. Many of them were found in the EGL and in the nascent WM (Fig. 12d, e). A few pH3-positive cells could also be seen in the IGL and the DCN (Fig. 12d). Within the nascent WM, pH3-positive nuclei could be found up to the tips of individual lobules (Fig. 12e). They were characterized by different staining intensity and patterns (i.e., with different levels of chromatin condensation [27] (Fig. 12f).

In p6 mice (Fig. 12g), pH3-positive cells were distributed similarly as on p4. However, by now, the EGL was clearly the area that contained the highest density of pH3-positive cells. Still, pH3-positive cells were also detectable within the central and lobular WM. Again, pH3-positive nuclei showed variable staining intensity and patterns.

At p8 (Fig. 12h), numbers of pH3-positive cells had much decreased compared with p6. pH3-positive cells were still detectable in the EGL of all cerebellar lobules, but in a lower density than at p6. Within the WM, only a few pH3-positive cells were found at this age.

At p15, only very few solitary pH3-positive cells were found in what remained of the EL, and also within the WM. In 28-day and adult mice, no pH3-positive cells could be detected within the cerebellum (not shown).

Figure 12 shows costaining for Pax2 and pH3. With a rare exception (Fig. 12c), Pax2 cells were negative for pH3. No obvious spatial association of Pax2 and pH3 cells could be observed at any age.

### Expression of the Neurotrophin Receptor, p75^NTR^, During Cerebellar Maturation

Staining for the neurotrophin receptor, p75^NTR^, was used with the intention to identify potential sonic hedgehog signaling [28].

**Fig. 13:**
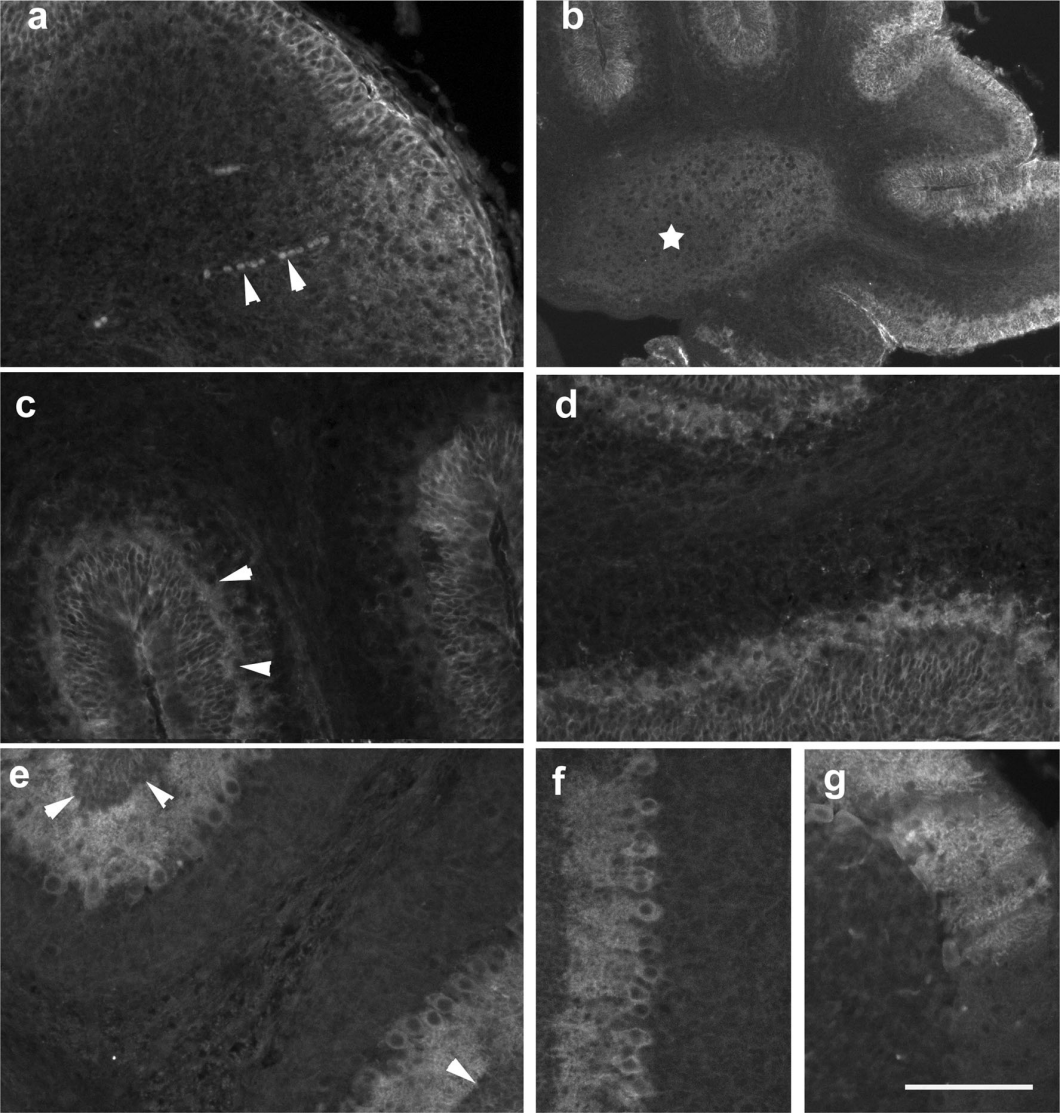
p75^NTR^ immunoreactivity in the developing cerebellum. **a** At p0, only the EGL is immunopositive for p75^NTR^. Note that cell nuclei are immunonegative, resulting in a web-like staining pattern. Within the prospective WM (visible in the lower left part of the image), no p75^NTR^-stained structures are visible. Roundish, blood-vessel associated, p75^NTR^-positive structures are marked by arrowheads. **b**, **c** Four days later, p75^NTR^ staining is strongest in the EGL. A somewhat diffuse staining can also be seen within the PCL. Nuclei of Purkinje cells are immunonegative (arrowheads in **c**). Immunonegative nuclei also stand out within the rather homogeneously p75^NTR^-stained DCN (marked by a star in **b**). The nascent WM is p75^NTR^-immunonegative. Panel **c** shows the basis of lobule 4/5. **d** At p6, p75^NTR^-staining is strongest in Purkinje cells, and again clearly visible in the EGL. In contrast, the developing WM remains immunonegative. Exemplary view of the basis of lobule 4/5. **e**, **f** At p8, Purkinje cells, especially in the medial lobules, still show strong staining for p75^NTR^, while staining in the EGL (visible along the left margin in panel **f**; and marked by arrowheads in **e**) has become weaker than at p6. As at younger ages, the WM remains immunonegative. **e** shows the basis of lobule 4/5; **f** shows an area of lobule 6/7. **g** At p15, only Purkinje cells in discrete areas of the medial lobules remain immunopositive for p75^NTR^. The transition between strongly stained and immunonegative cells is rather well demarcated. Scale bar (in panel **g**) = 80 μm for panels **a**, **c**–**g** and 210 μm for panel **b**

At p0, p75^NTR^ immunostaining was confined to the EGL (Fig. 13a). Cell nuclei were immunonegative, such that a web-like staining pattern resulted. Within the WM, no staining was visible, with the exception of a few blood-vessel associated, roundish structures that were also very intensely and homogeneously stained for Hoechst (Fig. 13a).

At p4, staining of the EGL of all cerebellar lobules appeared stronger than at p0. By now, also the PCL was p75^NTR^ immunopositive (Fig. 13b, c). In the PCL, staining was somewhat diffuse, but the large size of Purkinje cells allowed localizing p75^NTR^ in their cytoplasm surrounding immunonegative nuclei (Fig. 13c). A similar staining pattern was also visible in the DCN (Fig. 13b). No p75^NTR^-positive structures could be detected in the prospective WM (Fig. 13b, c) at p4.

At p6, p75^NTR^ immunoreactivity had generally increased, but the overall pattern of staining was identical to that described for p4. Purkinje neurons and the EGL were immunopositive in all cerebellar lobules (Fig. 13d), as were cells in the DCN (not shown). By now, delineation of individual cells based on their p75^NTR^ staining was clear-cut, particularly for Purkinje cells. No distinct p75^NTR^ immunosignal could be detected within the WM (Fig. 13d).

At p8, p75^NTR^ staining of the EGL had become weak, while Purkinje cells were still strongly p75^NTR^ immunopositive, particularly in the medial lobules (Fig. 13e, f). Purkinje cell staining in the anterior and posterior lobules was somewhat weaker. Again, no well localized p75^NTR^ immunosignal could be detected in the WM (Fig. 13e).

**Fig. 14:**
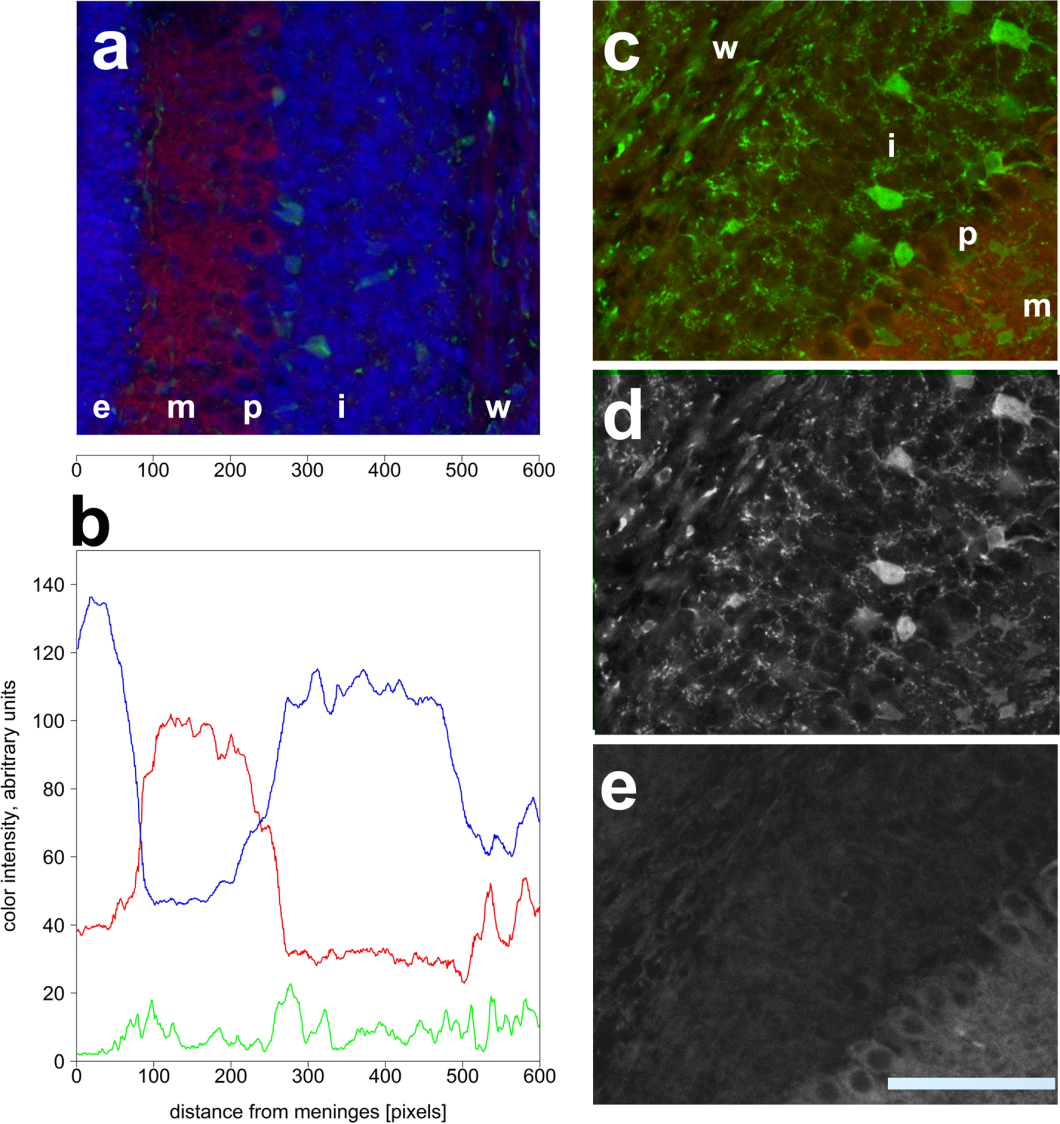
Quantitative definition of p75^NTR^ immunoreactivity in the cerebellar cortex. **a** Area from lobule 4/5 of a p8 cerebellum stained for p75^NTR^ (red), Pax2 (green) and nuclei (Hoechst, blue). Cortical layers are marked as follows: e, EGL; m, ML, p, PCL, i, IGL; and w, (prospective) WM. Numbers underneath the panel indicate positions from the EGL to the WM in pixels (for comparison with panel **b**). **b** Density scan of the image shown in panel **a**. **c**–**e** High power view from lobule 4/5 stained for Pax2 (green) and p75^NTR^ (red). Individual channels are shown in **d** (Pax2) and **e** (p75^NTR^). Note that no p75^NTR^ signal can be detected in or close to areas positive for Pax2, neither in the IGL (i) nor in the nascent WM (w). Scale bar (in panel **e**) = 80 μm in panels **a**, **c**–**e**

To better distinguish between true immunosignals and those due to nonspecific background staining, notably in Pax2 cells, we resorted to a (semi-) quantitative analysis. This was done using sections from p8 animals, since at this age, various layers can be well separated, and Pax2 cells may be found in all of these layers except the EGL. We selected areas from lobules 4/5 in which the borders between individual layers run essentially parallel. This allowed to average staining intensity along lines of pixels from the white matter to the EGL that transected layers perpendicularly. For each section analyzed, some 500–600 of such line scans were averaged, such that they may be interpreted as a profile of staining intensity. A typical image is shown in Fig. 14a, along with the resulting density scan (Fig. 14b) for p75^NTR^. This may be contrasted to the density of Pax2 cells, measured by GFP expression levels, and the average cell density, as assessed by nuclear staining. This approach confirmed that p75^NTR^ expression is essentially limited to Purkinje cells and cells in the EGL, whereas expression levels in the nascent IGL and in the nascent WM are very low. Indeed, focusing on the channel representing the p75^NTR^ immunosignal (red; shown in isolation in Fig. 14e) does not suggest any particular cellular structures enriched for p75^NTR^ immunoreactivity in these layers. This is in clear contrast with the “perinuclear” staining seen in the EGL. Significantly, we could not detect any p75^NTR^ signal (above background staining) in areas occupied by Pax2-GFP-positive perikarya, neither in the IGL nor in the nascent WM (compare Fig. 14, panels d and e).

At p15, p75^NTR^ is only detectable in Purkinje cells of the medial lobuli (Fig. 13g). There, areas with intensely p75^NTR^-positive cells contrasted with unstained areas. The transition between p75^NTR^-positive and p75^NTR^-negative Purkinje cells within these lobules was marked by a sharp border (Fig. 13g). In 28-day-old and adult mice, no p75^NTR^-positive structures could be found in the cerebellum (not shown).

Exemplary views from costaining for Pax2 and p75^NTR^ are shown in Fig. 14. We could not detect any Pax2 cell positive for p75^NTR^, at any age; nor could we detect p75^NTR^-positive structures in the WM showing any association with Pax2 cells.

## Discussion

We compared the developmental distribution, in the early postnatal cerebellum, of Pax2-defined precursors of cerebellar inhibitory interneurons with that of cerebellar macro- and microglial cells. We also analyzed the distribution of proliferating cells and cells expressing the neurotrophin receptor, p75^NTR^, during this developmental period. Our results document that early-migrating Pax2 cells reach the cerebellar cortex well before most of the (glial) maturation markers analyzed are expressed in the cerebellar anlage. As development proceeds, migrating precursors of cerebellar inhibitory interneurons have to navigate an environment that is increasingly populated by maturing glia.

The time course of Pax2-GFP expression and the distribution of Pax2 cells within the early postnatal cerebellar anlage described here is consistent with previous results [9, 11] and underlines the utility of this marker to follow the development of cerebellar inhibitory interneurons. The one exception here is the presence of Pax2-GFP-positive cells within the meningeal surface (visible in Fig. 2, panels a, b). The nature of these cells is unknown, although their appearance suggests that they might be vascular cells. It has been suggested that endothelial cells might express Pax2 [29]. While there is no independent evidence that the transgene used here is expressed in vascular cells [11, 18], we note that this would not affect the interpretation of our findings. Pax2-GFP-positive cells within the cerebellar anlage may be faithfully considered precursors of inhibitory interneurons.

CNP and MBP are two established markers to follow oligodendrocyte development, with CNP as the earliest known myelin-specific protein expressed by cells of the oligodendrocyte lineage [19, 20]; in contrast, MBP becomes detectable immediately before formation of the myelin sheath begins [19, 23], and thus indicates the final differentiation of these cells. The spatio-temporal pattern of expression of both of these markers indicates that, as previously documented in chicken [30], and surmised for rat [24, 31], murine cerebellar oligodendrocytes also immigrate into the cerebellar anlage through the velum medullare (superius). From there, they disperse throughout the cerebellar anlage, except into the layers above the PCL, within about 8 days.

The differential time course described above for the appearance and distribution of Pax2 cells and the oligodendrocyte differentiation markers, CNP-1 and MBP, clearly indicate that many Pax2 cells reach the cerebellar cortex prior to the arrival/differentiation of CNP-1- and/or MBP-defined oligodendroglia in the cerebellar anlage, i.e., prior to about p4/6. Consequently, these cells are not exposed to CNP-1 and/or MBP-defined oligodendrocytes during their migration and early settling. In contrast, Pax2 cells traversing the nascent WM after p4/6 navigate a territory characterized by increasing density of CNP-1- and/or MBP-positive cells and extensively mingle with them (for p4/6 see Fig. 7a; for p8, Fig. 7b–e).

Comparison of the distribution of these oligodendrocyte maturation markers with that of Pax2 cells, notably at p8 (Fig. 2 and Figs. 4 and 5; Fig. 7b–f for double labeled sections), documents that increasingly mature glial cells do not preclude the migration of Pax2 cells. Nor do they seem to “push out” Pax2 cells from the maturing WM. This is also confirmed by the extensive mingling of Pax2 cells and CNP and MBP-positive glia seen in double-stained sections (Fig. 7b–f).

The ablation of cerebellar oligodendrocytes during the first postnatal week, but not later, has been shown to result in extensive loss of basket and stellate cells [32]. Results by these authors also suggest that the loss of oligodendrocytes might cause a premature emigration of interneuronal precursors from the nascent WM, and possibly their premature differentiation in the IGL. In addition, it is known that myelinating oligodendrocytes powerfully inhibit neurite growth [33]. Together, these findings suggest that oligodendrocytes inhibit the (premature) exit of Pax2 cells and their differentiation, specifically of those Pax2-positive interneuron precursors that transit the nascent WM for protracted periods. Previous experiments (cf [34] and references given there) have established that Pax2 cells follow an internal clock, set in fact by the expression of Pax2. The present findings, together with the evidence obtained from oligodendrocyte-ablation studies (cf above), suggest/indicate that maturing cerebellar glia can reset this clock.

Whereas CNP-1 is a marker that allows following the invasion of prospective oligodendrocytes into the cerebellar anlage, GFAP is certainly not a marker to do the same for all cells that will eventually express this protein. If we may consider, for the moment, all GFAP-positive cells as one group, we may again note that many Pax2 cells reach the ML well before GFAP is expressed.

Expression of GFAP in the various regions of (prospective) WM shows considerable temporal and regional differences, and consequently, so does the mingling of GFAP-positive and Pax2 cells. If Pax2 cell differentiation was affected by GFAP-positive cells in the WM, one might expect also regional differences in terminal differentiation of Pax2 cell–derived interneurons. Such differences have so far not been observed.

On the other hand, it is well established that cerebellar gray matter regions that are populated by distinct types of inhibitory interneurons are also characterized by strikingly different types of astroglia, thus as Bergman glia in the ML, and velate and protoplasmic astrocytes in the IGL (see [35, 36] and [37] for detailed discussion). The yet distinct character of astrocytes in the DCN is again supported by our finding that their hallmark characteristic, i.e., the lack of GFAP expression [38], may be observed throughout their development.

Thus, while the expression pattern of GFAP in the nascent WM does not suggest that GFAP-defined cells differentially affect the fate of Pax2 cells in transit, the local terminal differentiation of Pax2 cells may well be influenced by the distinct types of astrocytes found in the ML, the IGL, or in deep nuclei.

Of all differentiation/cell-type markers analyzed here, Iba-1 is the only one expressed as early as p0 in the nascent white matter. Also, the density of Iba-1 immunoreactive structures decreased with ongoing cerebellar maturation, and their morphology changed from roundish, amoeboid to ramified, as typical for mature microglia. These latter cells are well known to continuously and actively monitor their environment (cf [39]). Roundish/amoeboid microglia persisted, in the early postnatal cerebellum, preferentially in the distal lobular WM and the IGL.

The distribution and changing morphology of microglia observed presently is fully consistent with the descriptions given by Ashwell et al. [40], by Perez-Pouchoulen [15], and again by Nakayama and colleagues [41]. The temporal changes in microglial density and morphology observed presently support the scenario suggested by Nakayama [41] that microglia invade the cerebellar anlage primarily via the nascent WM.

As the shape of Iba-1-positive cells is an indicator of microglial maturity or activation (cf [41]; see also [42–44]), this implies that Pax2 cells reaching the cerebellar cortex early on would be confronted/exposed only to immature (roundish) Iba-1 cells. Pax2 cells that traverse the WM later on also encounter more mature (ramified) microglia.

The simultaneous presence of microglia and migrating Pax2 cells in the IGL and the nascent ML from day 6/8 onwards is particularly intriguing. On the one hand, it has recently been shown that Pax2 cells en route through these layers form transient synapses that direct their migration. On the other hand, microglia are known to modify or eliminate supernumerary synapses (for a review, see [45]). Lastly, Nakayama et al. [41] recently presented data indicating that elimination of microglial ablation resulted in a reduction of GABAergic transmission in the developing cerebellar cortex due to a presynaptic deficiency (i.e., insufficient signaling from basket/stellate cells). Eventually, this resulted also in a reduction of ML thickness [41]. The present findings beg the question whether this might also be due to hindered migration of basket/stellate cell precursors following microglial ablation.

We would also like to draw attention to correlation between the morphological differentiation of microglia and the appearance of GFAP-positive cells. This is best seen in lobular tips, where roundish microglia can be seen first, followed by the appearance of GFAP-positive cells, again followed by a switch of microglia to a ramified morphology. Such a temporal sequence has also been observed in the hippocampus [46]. Indeed, both the proliferation of astroglial precursors and their maturation (GFAP-expression) are stimulated by microglia [47–49]. Conversely, it has been suggested that differentiated astrocytes control microglia maturation [44, 50–52]. This exemplifies that an understanding of Pax2 cell maturation will eventually also require an appreciation of how its various cellular interaction partners may influence each other (cf also [53]).

While the signals by which glial and microglial cells may affect migration and maturation of Pax2 cells remain enigmatic for the moment, Purkinje-cell-derived sonic hedgehog (Shh) is an obvious candidate that might impinge on Pax2 cells in transit through the white matter. Fleming et al. [12] have previously shown that proliferation of precursors of both glial and Pax2 cells (or rather, their precursors) in the prospective WM is sensitive to Shh, as verified by a Gli1-based transgene. Induction of p75^NGF^ was recently identified as another readout for Shh signaling in granule cells [28].

Whereas we could detect an unambiguous p75^NTR^ signal in those regions of the cerebellum where it had been described before, viz., proliferative, Shh-sensitive granule cell precursors [28] and, from p4 onward, in Purkinje cells [54], we could not detect Pax2 cells that also stained for p75^NTR^. The few very intensely p75^NTR^-stained structures in the prospective WM also showed strong and homogenous nuclear staining for Hoechst, suggesting/indicating that these are apoptotic cells, possibly aberrant granule cells that have been suggested to be eliminated by MAG/p75^NTR^-triggered apoptosis [55]. They also differed from p75^NTR^-positive granule cell precursors and Purkinje cells in that the p75^NTR^ signal completely colocalized with their condensed nuclei.

Together, these findings indicate that precursors within the prospective WM that will eventually mature and populate the cerebellum do not express p75^NTR^, at least not at levels detectable by immunostaining. Moreover, as Pax2 cells, like their Pax2-negative precursors, are sensitive to Shh [56], this implies that Shh-signal processing in these cells diverges from that in granule cell precursors. Unfortunately, the technical tools to identify canonical Gli1 expression at the cellular level are not yet available to follow up on this issue. Finally, these findings also suggest that precursors in the WM may not give origin to p75^NTR^-positive medulloblastoma [57], though more direct evidence would be required to strengthen this idea.

To monitor the mitotic activity of Pax2 cells, or their spatial relationship with proliferating cells, we stained sections for phosphohistone H3. The fact that we found only very rare Pax2 cells that also stained for pH3 fully agrees with previous findings that expression of Pax2 essentially starts in postmitotic precursors of inhibitory cerebellar interneurons (Pax2 cells) [10, 11, 56]. Further, we could not identify any spatial correlation between Pax2 cells and proliferating cells. The one exception of this is the well-known exclusion of Pax2-positive cells from the proliferative active EGL.

## Conclusions

The present study relates the formation and migration of Pax2-defined precursors of cerebellar inhibitory interneuron to the neurochemical maturation and dispersion of cerebellar glia, including microglia. Pax2 cells traversing the nascent WM at different times are confronted with a progressively changing complement of macro- and microglial cells, and might be exposed to dynamically changing signals emanating from these cells. While the present findings do not inform us as to the nature of the signaling molecule(s) that in fact can even reprogram heterochronically transplanted Pax2 cells to the hosts laminar fate [10], they narrow down likely sources of these developmental regulators. They also suggest that signaling molecules differentially affecting Pax2 cells that reach the cerebellar cortex at different developmental stages should be coregulated with glial differentiation markers such as the ones studied here. Finally, the continued intermingling of Pax2 cells and various types of glia in the nascent white matter throughout development indicates that maturing glia do not provide Pax2 cells with a signal that triggers their exit from the white matter.
